# Negative regulation of seed germination by maternal AFB1 and AFB5 in Arabidopsis

**DOI:** 10.1042/BSR20221504

**Published:** 2022-09-12

**Authors:** Yixing Wang, Nadjeschda J. Goertz, Emily Rillo, Ming Yang

**Affiliations:** Department of Plant Biology, Ecology, and Evolution, Oklahoma State University, 301 Physical Sciences, Stillwater, OK 74078, U.S.A.

**Keywords:** auxin sensitivity, auxin signaling, chalaza, funiculus, maternal effect, seed dormancy

## Abstract

The plant hormone auxin suppresses seed germination, but how auxin does it remains poorly understood. While studying the functions of the AUXIN SIGNALING F-BOX (AFB) auxin co-receptors in Arabidopsis, we consistently isolated AFB1 and AFB5 in reproductive tissues in co-immunoprecipitation experiments using their interacting protein ASK1 as the bait. However, T_2_ seeds of the *AFB1* or *AFB5* transgenic lines generated for the co-immunoprecipitation experiments frequently failed to germinate, which led to the studies of seed germination in these plants and *afb1* and *afb5* mutants, and *AFB1* and *AFB5* expression in nearly mature fruit and imbibed seeds using *AFB1:GUS* and *AFB5:GUS* lines. We found that AFB1 and AFB5 acted in maternal tissues to suppress seed germination and their effects were positively correlated with the plants’ sensitivity to indole acetic acid. Conversely, *afb1* and *afb5* single mutants exhibited faster seed germination than the wild type and the seeds of the *afb1-5afb5-5* double mutant germinated even faster than those of the *afb1-5* and *afb5-5* single mutants. Seed germination of the *afb1-5afb5-5* double mutant also exhibited higher sensitivity to gibberellic acid than that of the wild-type and the *afb1-3, afb1-5 and afb5-5* single mutants. Both *AFB1* and *AFB5* were expressed in the funiculus during seed maturation, and *AFB1* was also transiently expressed in a small chalazal region surrounding the hilum in the seed coat during seed imbibition. Therefore, AFB1 and AFB5 likely suppress seed germination in the funiculus and AFB1 also briefly suppresses seed germination in the chalaza during seed imbibition.

## Introduction

Maintaining or breaking seed dormancy in appropriate environmental conditions is crucial to survival of plants. The molecular mechanism governing seed dormancy regulation is very complex as many proteins of diverse functions and multiple hormones have been reported to affect seed dormancy. However, current understanding of seed dormancy regulation has not been established at the systems level. A systems-level understanding of seed dormancy regulation requires identification of all the major regulators of the seed germination process, including those acting in hormonal signaling pathways.

Hormonal regulation of seed dormancy has been most extensively studied with the plant hormones abscisic acid (ABA) and gibberellic acid (GA). ABA is known for promoting seed dormancy whereas GA for breaking seed dormancy. The ABA and GA signaling pathways interact with each other, with the ABA pathway negatively regulating the GA pathway and the GA pathway both negatively and positively regulating the ABA pathway [1]. The ratio between the concentrations of ABA and GA in the seed is a major parameter for determining whether the seed is dormant or not [1,2]. The auxin signaling pathway also interacts with the ABA signaling pathway to promote seed dormancy [3–7]. The major theme here is that auxin signaling activates the functions of ARF10/16 that further induce ABI3 expression. ABI3 is a major component of the ABA signaling pathway for seed dormancy. However, the understanding of how auxin signaling regulates seed dormancy is still at an early stage. Even the genetic networks involving ABA and GA signaling in seed dormancy regulation are still sketchy.

A mature angiosperm seed has a seed coat that surrounds the endosperm (diminished in some species) that surrounds the embryo. The seed coat develops from maternal tissues that typically include the integuments and the chalaza of the ovule [8,9]. Any part of the seed can be involved in establishing seed dormancy, but the seed coat is responsible for the maternal control of seed dormancy. Studies in diverse species have established that the seed coat exerts negative physiological effects on germination [10–16]. Currently, which auxin signaling components act in the seed coat for dormancy regulation is unclear.

The auxin signaling mechanism was largely elucidated in the model plant Arabidopsis with the characterization of the Skp1-Cullin-ARABIDOPSIS AUXIN SIGNALING F-BOX (SCF^AFB^) ubiquitin ligases and their downstream AUX/IAA and ARF proteins. The AFB proteins in the SCF^AFB^s bind both auxin and members of the AUX/IAA protein family, which leads to the ubiquitination and degradation of the AUX/IAA proteins [17–22]. Degradation of AUX/IAAs frees their other interacting proteins, namely the transcriptional regulators AUXIN RESPONSE FACTORs (ARFs) that then trigger transcriptional changes in many downstream genes [17,20,23–25]. Both the AFB protein and AUX/IAA protein act as auxin co-receptors and the auxin molecule serves as a glue to enhance the interaction between the AFB protein and the AUX/IAA protein in an SCF^AFB^-AUX/IAA complex [19].

The general biological functions of the AFBs are mostly understood through genetic studies of *afb* mutants. Single null mutants of AFBs either do not exhibit a mutant phenotype or exhibit a mild mutant phenotype while increasing the number of *afb* mutant loci in higher-order mutants leads to increasingly severe mutant phenotypes [26–28]. These mutant plants are also insensitive to external auxin treatment [27]. In comparison with the loss-of-function phenotypes, overexpression of an AFB can increase plants’ sensitivity to auxin and cause mild defects [29,30]. To date, no single *afb* mutants have been shown to have a seed germination defect although faster-than-normal seed germination has been reported for the *tir1afb2* and *tir1afb3* double mutants [5]. Here, we report that AFB1 and AFB5 synergistically act in maternal tissues to suppress seed germination in Arabidopsis. Our findings provide insight into how auxin signaling is involved in seed dormancy regulation.

## Results

### AFB1 and AFB5 act synergistically in suppressing seed germination

**Figure 1:**
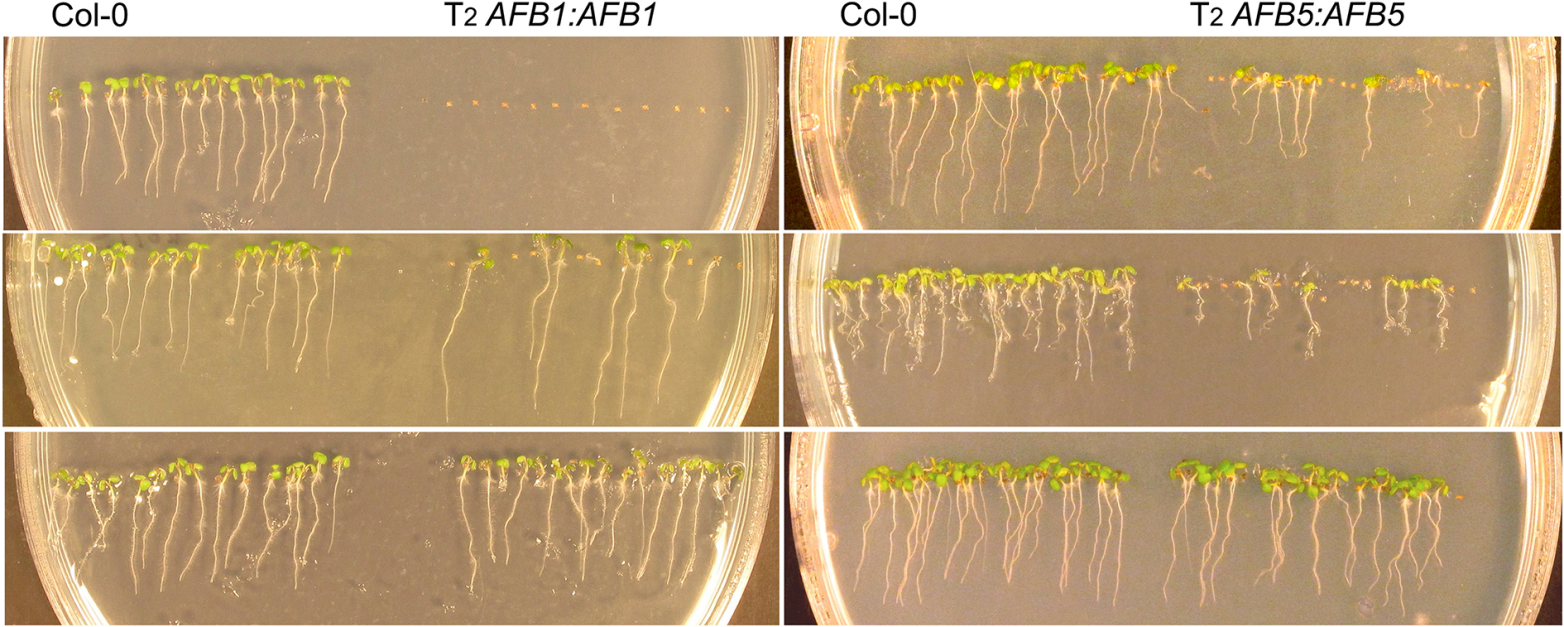
Seed germination on the MS agar medium Shown were three independent *AFB1:AFB1* T_2_ lines (left) and three independent *AFB5:AFB5* T_2_ lines (right) along with the wild-type control (Col-0). The photos were taken 5 days after seed sowing.

**Figure 2:**
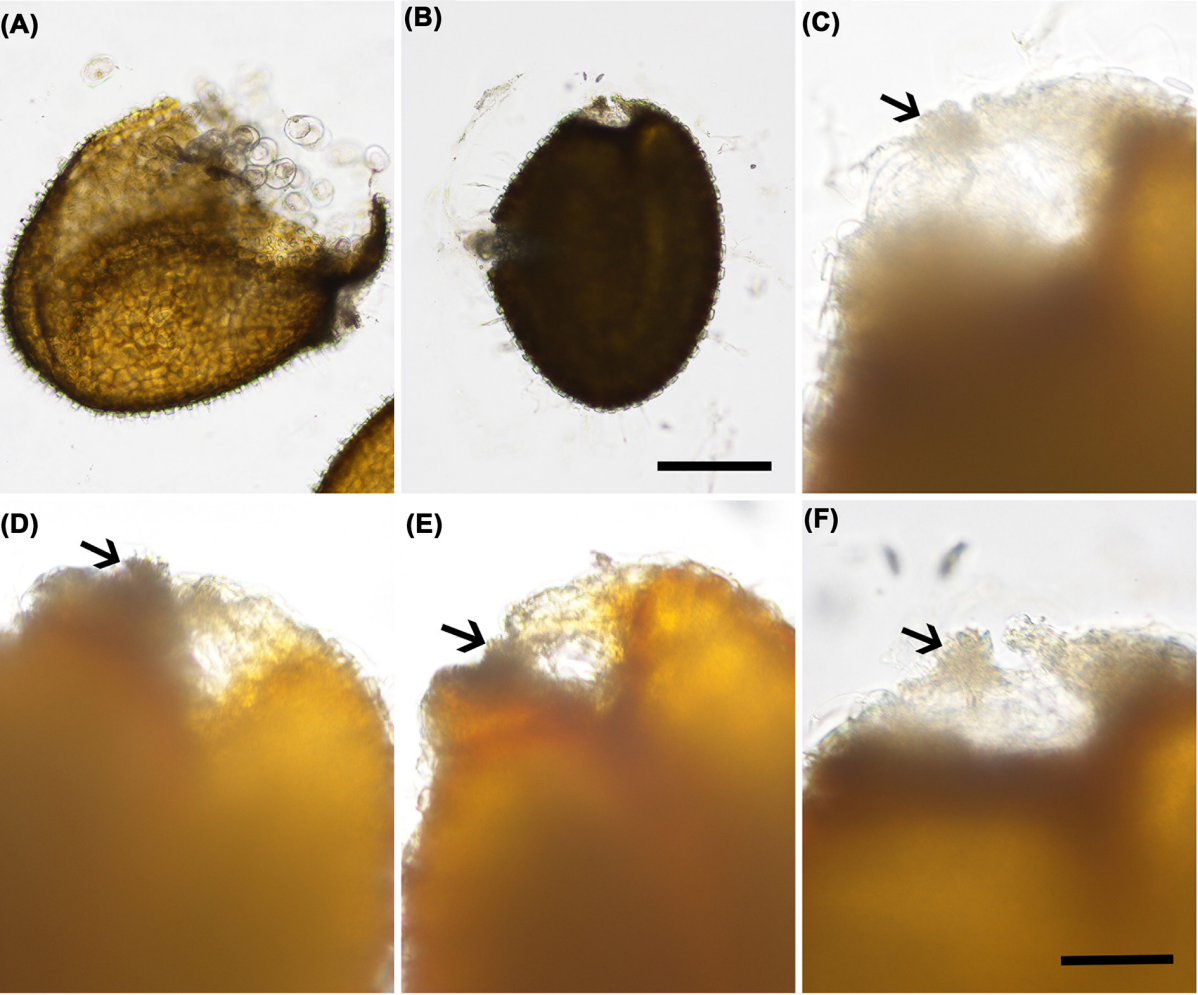
Comparison of seed morphology between germinated and ungerminated *AFB1:AFB1* seeds (**A–C,F**) 20 days on the MS agar medium. (**D,E**) Approximately 5 min in water. Arrows indicate the hila. (A) Seed coat from a germinated seed. (B) An ungerminated seed. (C) A close-up view of an erected transparent parenchyma region of the seed coat continuous with the hilum from the seed in (B). (D,E) For comparison with (C), the parenchyma region continuous with the hilum had not erected with the short period of imbibition. (F) A seed with the erected parenchyma region similar to that in (C). Bar in (B) for (A) and (B) = 200 µm, and bar in (F) for (C-F) = 50 µm.

In the process of investigating the functions of SCFs in Arabidopsis, we consistently identified AFB1 and AFB5, not other AFBs, as ARABIDOPSOS SKP1-LIKE1 (ASK1)-interacting proteins in inflorescences by co-immunoprecipitation (Co-IP) followed by liquid chromatography with tandem mass spectrometry (LC-MS/MS) in four independent experiments (Supplementary Table S1). AFB1 and AFB5 thus were likely more abundant and/or more stable than other AFBs in the reproductive tissues. To investigate the roles of AFB1 and AFB5 in reproductive development, we generated transgenic plants harboring an *AFB1* and an *AFB5* transgene driven by either its native or the *ASK1* promoter in the *afb1-3* and *afb5-5* backgrounds, respectively. Using *afb1-5* and *afb5-5* in these experiments was to eliminate or reduce the interference of the endogenous AFB1 or AFB5 in subsequent analyses. We frequently found that the T_2_ seeds either could not germinate at all or germinated poorly 5 days after planting on the agar medium (Table 1 and Figure 1). The ungerminated seeds remained ungerminated even after 20 days on the agar medium, even though they were highly imbibed with a broken seed coat (Figure 2). Clearly the transgenes had a profound effect on seed germination. These observations were consistent with Liu et al.’s [5] findings showing auxin signaling positively regulates seed dormancy.

**Table 1:** Numbers of transgenic lines with defective T_2_ seed germination and the total numbers of transgenic lines examined

Transgene	Number of defective lines/number of lines examined
*AFB1:AFB1*	13/19
*ASK1:FLAG-AFB1*	0/4
*AFB5:AFB5*	5/8
*ASK1:AFB5*	3/3

**Figure 3:**
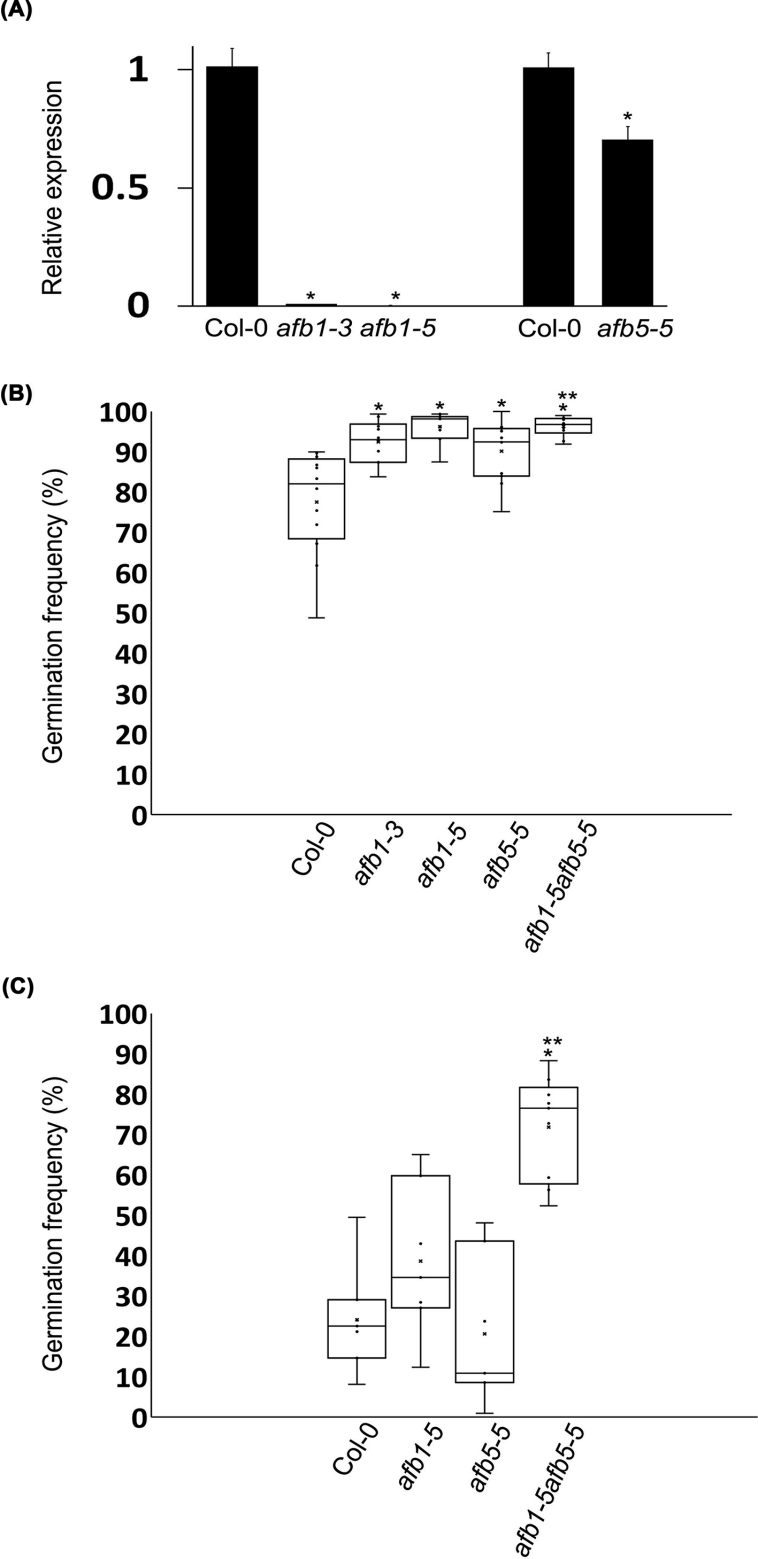
Relative expression levels of *AFB1* and *AFB5* and seed germination frequencies (**A**) Shown are means ± standard errors. The expression level in Col-0 is defined as 1. (**B**) Box-and-whisker plot of seed germination frequencies after 72-h imbibition. (**C**) Box-and-whisker plot of seed germination frequencies after 58-h imbibition. The symbol ‘*’ denotes statistically significant differences when compared with Col-0 (*t*-test, *P* < 0.05). The symbol ‘**’ denotes statistically significant differences when compared with the single mutants (*t*-test, *P* < 0.05).

We next characterized three loss-of-function mutant alleles, namely *afb1-3*, *afb1-5*, and *afb5-5*. The *AFB1* transcript level in 2-week-old seedlings was essentially zero in *afb1-3* and *afb1-5* in comparison with that in the wild type, indicating that these mutants are likely null alleles (Figure 3A). The *AFB5* transcript level in *afb5-5* was approximately 70% of the wild-type level (Figure 3A). We then tested the seed germination frequencies of Col-0 and these mutants after 72-h imbibition on wet filter paper in a Petri dish. The *afb1-3*, *afb1-5*, and *afb5-5* mutants had average germination frequencies of 92.5%, 96.2%, and 90.1%, respectively (Figure 3B). All of them were statistically higher than the average germination frequency (77.6%) of Col-0 (*t*-test, 10 ≤ *n* ≤ 12, *P* < 0.05). We also generated the *afb1-5afb5-5* double mutant and found that its average germination frequency was 96.3% after 72-h imbibition, which was statistically higher than those of Col-0 and *afb5-5* but not different from that of *afb1-5* (*t*-tests, 10 ≤ *n* ≤ 11, *P* < 0.05). These results indicated that AFB1 and AFB5 normally inhibit seed germination.

The above experiment showed that the average germination frequency of the *afb1-5afb5-5* double mutant was higher than that of *afb5-5*, but whether it was also higher than that of *afb1-5* was unclear because the seeds of *afb1-5* and the double mutant already nearly fully germinated at the time of 72-h imbibition. To further investigate whether the average germination frequencies of *afb1-5* and *afb5-5* differ from that of *afb1-5afb5-5*, we scored germinated and ungerminated seeds of Col-0, *afb1-5*, *afb5-5*, and *afb1-5afb5-5* using the above filter paper germination assay with a 58-h imbibition treatment. This experiment produced average germination frequencies of *afb1-5* and *afb5-5* at 38.6% and 20.7%, respectively, neither of which was statistically different from Col-0’s 24.2% (Figure 3C; *t*-test, *n* = 7, *P* > 0.05). However, the average germination frequency of *afb1-5afb5-5* was 71.9%, which was statistically higher than those of Col-0 and the single mutants (Figure 3C; *t*-test, 7 ≤ *n* ≤ 9, *P* < 0.05). Moreover, at the time of 58-h imbibition, the large increase in the germination frequency of *afb1-5afb5-5* could not be simply accounted for by the addition of the single mutants' effects on germination, suggesting that the *afb1-5* and *afb5-5* mutations synergistically promoted seed germination in the *afb1-5afb5-5* double mutant.

### The *afb1-5afb5-5* double mutant is more sensitive to gibberellic acid than the wild-type and *afb1* and *afb5* single mutants in seed germination

**Figure 4:**
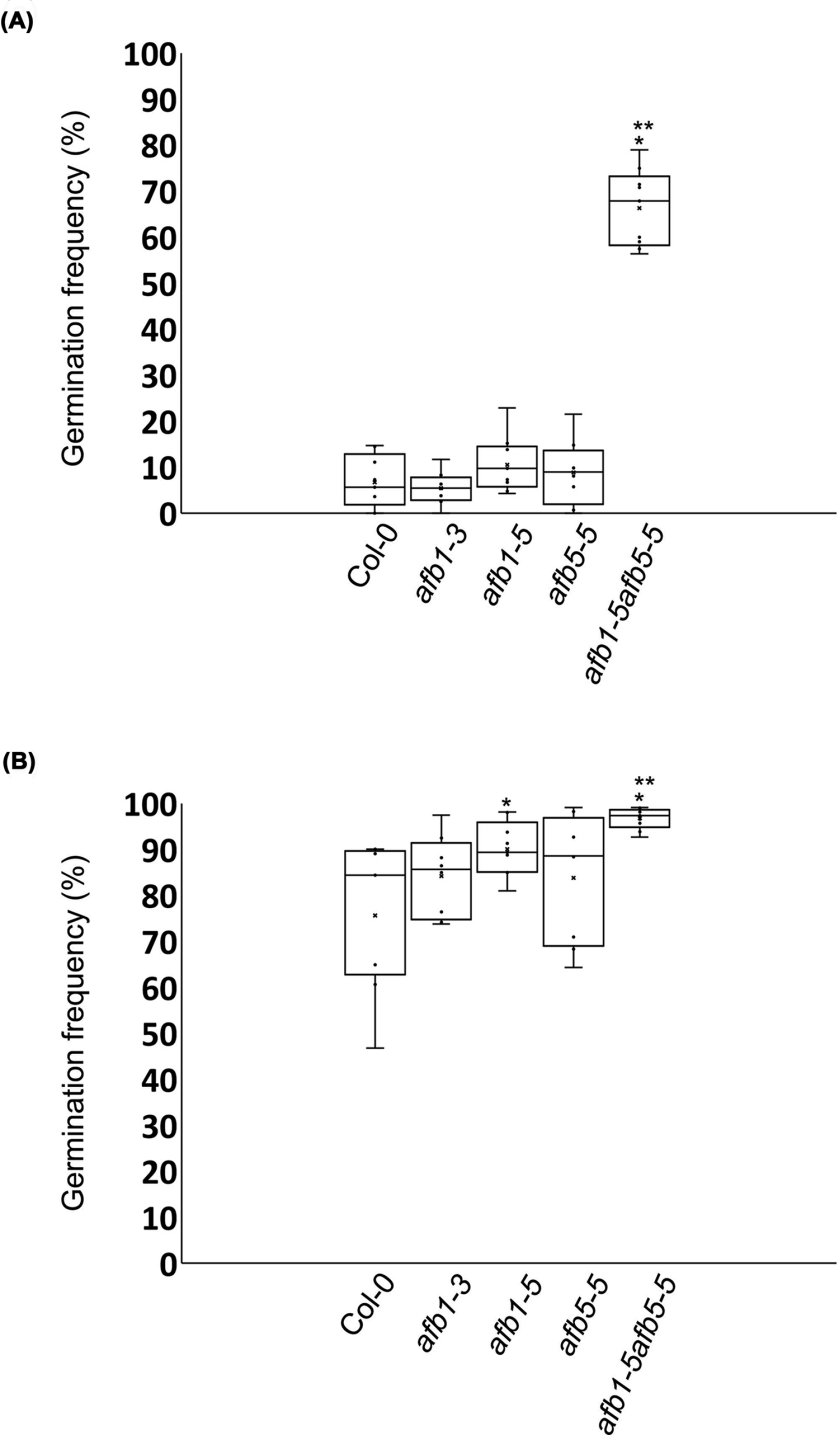
Germination frequencies of seeds treated with 1 µM GA (**A**) Box-and-whisker plot of seed germination frequencies at the time of 46-h imbibition. (**B**) Box-and-whisker plot of seed germination frequencies at the time of 64-h imbibition. The symbol ‘*’ denotes statistically significant differences when compared with Col-0 (*t*-test,* P* < 0.05). The symbol ‘**’ denotes statistically significant differences when compared with the single mutants (*t*-test, *P* < 0.05).

GA is known to promote seed germination and its effect on seed germination may be more pronounced when auxin’s negative effect on seed germination is reduced. To test this possibility, we replaced water with 1 µM GA in the seed germination assay as described for Figure 3B, except that the incubator temperature was reduced to 20°C and the germinated and ungerminated seeds were counted at the times of 46- and 64-h imbibition. The reductions in temperature and imbibition duration were to ensure that the seeds were counted between an early germination stage and the full germination stage and to attain the germination frequencies of the *afb1-5afb5-5* double mutant comparable to those in Figure 3C,B, respectively. The average germination frequencies after the 46-h imbibition with the GA3 solution were 6.7%, 5.4%, 10.5%, 8.8%, and 66.3% for Col-0, *afb1-3*, *afb1-5*, *afb5-5*, and *afb1-5afb5-5*, respectively (Figure 4A). The average germination frequency of *afb1-5afb5-5* was higher than those of the wild type and the single mutants (Figure 4A; *t*-test, *n* = 8 or 9, *P* < 0.05) while those of the single mutants were not statistically different from that of the wild type (Figure 4A; *t*-test, *n* = 8 or 9, *P* > 0.05). Such differences in the germination frequency were still evident among the genotypes at the time of 64-h imbibition although the average germination frequency of *afb1-5* was also higher than that of the wild type (Figure 4B; *t*-test, *n* = 8 or 9, *P* > 0.05). The average germination frequency of *afb1-5afb5-5* at the time of 46-h imbibition with the GA solution was nearly tenfold of that of the wild type (Figure 4A). In comparison, the average germination frequency of *afb1-5afb5-5* was only approximately 3-fold of that of the wild type at the time of 58-h imbibition with water (Figure 3C). It is noted that these two germination frequencies of *afb1-5afb5-5* only slightly differed from each other in this comparison (66.3% vs. 71.9%). Furthermore, Figure 3B and C and Figure 4 together showed that the seeds of the single mutants and the wild type had similar levels of sensitivity to GA. Therefore, the seeds of *afb1-5afb5-5* were much more sensitive to GA than the seeds of the wild type and the single mutants.

### AFB1 and AFB5 act in maternal tissues to regulate seed germination

**Figure 5:**
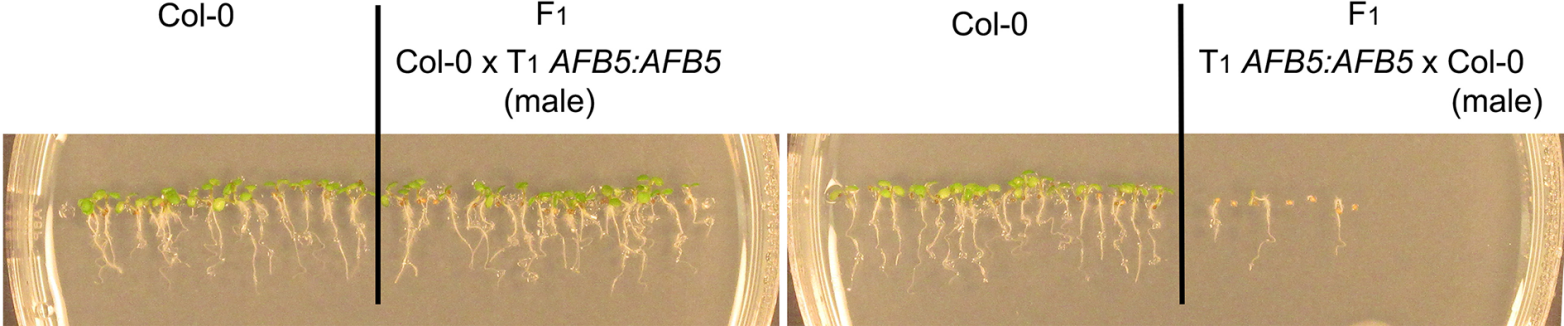
Germination of F_1_ seeds of reciprocal crosses between Col-0 and a T_1_ *AFB5:AFB5* line The photos were taken five days after seed sowing; Col-0, wild-type control.

The failure to germinate was 100% for the T_2_ seeds of three of the *AFB1:AFB1* lines (Figure 1). Considering that the T1 seeds apparently germinated to produce the T_2_ seeds and the T_2_ seeds should have segregated for the transgene in both the endosperm and the embryo, the complete non-germination phenotype in these lines indicated that the *AFB1* transgene caused the seed non-germination phenotype in the maternal tissues in the seed coat. None of the *AFB5* transgenic lines exhibited 100% T_2_ seed non-germination. However, the F_1_ seeds from crosses between carpels of an *AFB5:AFB5* T_1_ plant and pollen of a Col-0 plant, not F1 seeds from the reciprocal crosses between these two plants, exhibited a non-germination defect, indicating that the *AFB5* transgene also acted in the seed coat to suppress seed germination (Figure 5).

### Severity of seed non-germination phenotype is positively correlated with sensitivity to indole acetic acid

**Figure 6:**
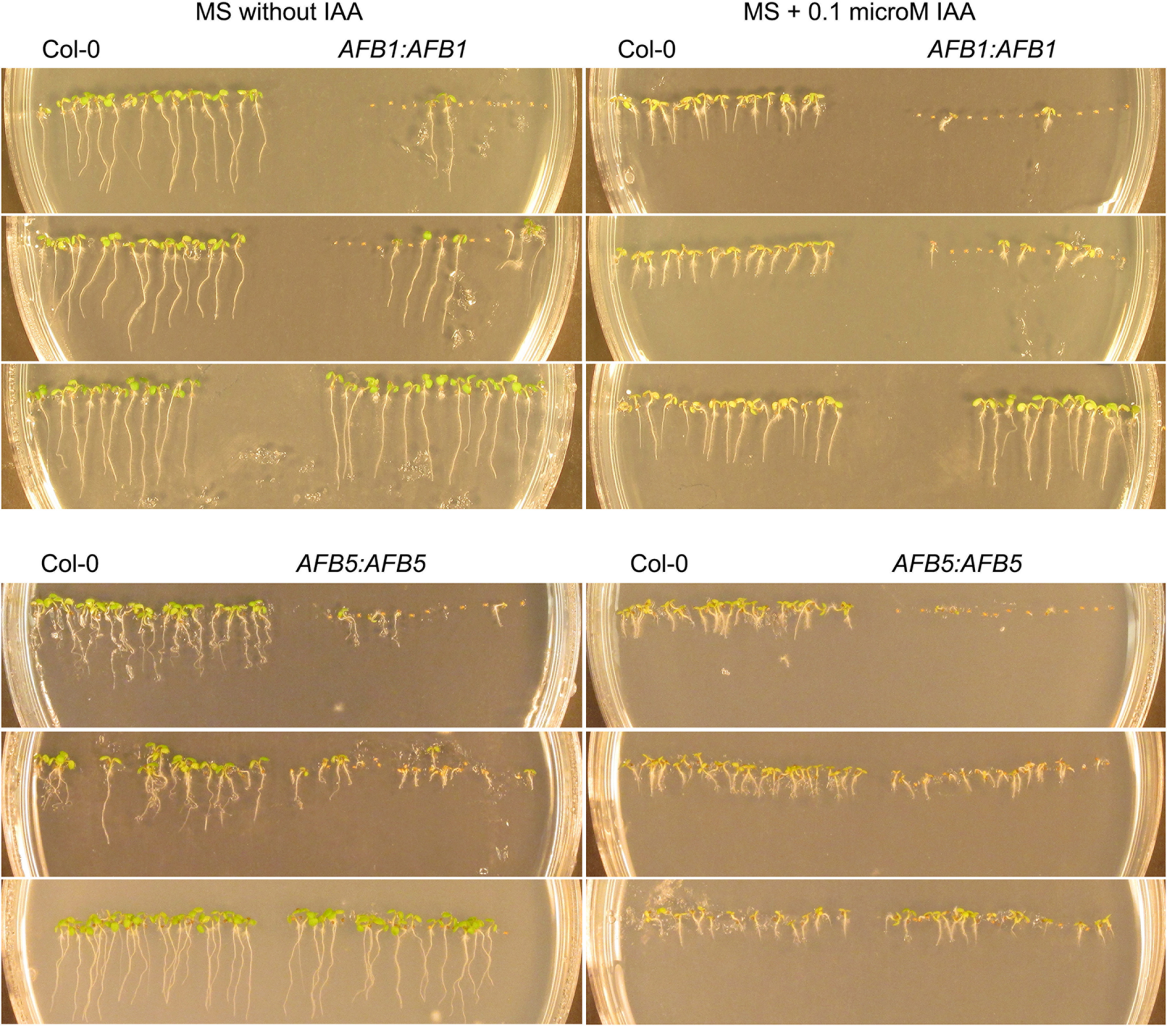
Negative correlation between IAA sensitivity and seed germination shown with representative *AFB1:AFB1* and *AFB5:AFB5* lines For each genotype with the IAA treatment, the top, middle, and bottom samples represent lines with three sensitivity levels to IAA, respectively. The overall results are summarized in Table 2. The corresponding samples without the IAA treatment serve as additional controls. The photos were taken 5 days after seed sowing.

If the above germination defect in the T_2_ seeds resulted from the effect of the transgene in the seed coat derived from the T_1_ plant, the transgene should segregate in the T_2_ plants and its expression levels in individual T_2_ plants would not always be correlated with the severity level of the corresponding T_2_ germination defect. Nonetheless, we predicted that at the population level, the T_2_ plants’ sensitivity to auxin should still be correlated with the corresponding T_2_ seed germination defect. To test this prediction, the T_2_ and Col-0 seeds were planted on the same agar medium plates with or without 0.01 µM indole acetic acid (IAA). The sensitivity of the transgenic plants to IAA were assessed based on their root lengths in comparison with the Col-0 root lengths on the same cultural plates. Shorter- and longer-than-control roots mean more and less sensitive to IAA than the control, respectively [31]. Figure 6 shows examples of germination phenotypes along with three levels of IAA sensitivity for the *AFB1*:*AFB1* and *AFB5:AFB5* lines when compared with Col-0: (1) severely defective germination, highly sensitive to IAA, (2) moderately defective germination, moderately sensitive to IAA, and (3) normal germination, wild-type-level sensitivity or insensitive to IAA (Table 2). The first two IAA-sensitivity levels indicated the full complementation, and the third IAA-sensitivity level full, partial, or no complementation, of the *afb1-3* or *afb5-5* mutation by the corresponding transgene. The results show that the auxin sensitivity levels in the transgenic lines were positively correlated with the severity levels of the seed germination defect and the transgenes complemented the *afb1-3* and *afb5-5* mutations in most of the transgenic lines, respectively.

**Table 2:** Assessment of seed germination and seedling sensitivity to IAA in *AFB1* and *AFB5* transgenic lines at the T_2_ generation

Line number	Seed germination	Sensitivity to IAA
***AFB1:AFB1***		
2-2	Defective	More sensitive
2-3	Normal	Less sensitive
3-1	Defective	More sensitive
3-3	Defective	More sensitive
3-4	Normal	Wild-type-like
3-5	Defective	More sensitive
4-2	Defective	More sensitive
5-1	Defective	More sensitive
5-6	Normal	Wild-type-like
5-7	Normal	Less sensitive
6-1	Defective	More sensitive
6-2	Defective	More sensitive
6-3	Defective	More sensitive
6-4	Defective	More sensitive
***AFB5:AFB5***		
1-4	Defective	More sensitive
1-5	Normal	Wild-type-like
2-7	Defective	More sentitive
3-3	Defective	More sensitive
4-5	Defective	More sensitive

### *AFB1* and *AFB5* are expressed in the funiculus in nearly mature fruit and *AFB1* is also transiently expressed in a seed coat region surrounding the hilum in postharvest seeds upon imbibition

**Figure 7:**
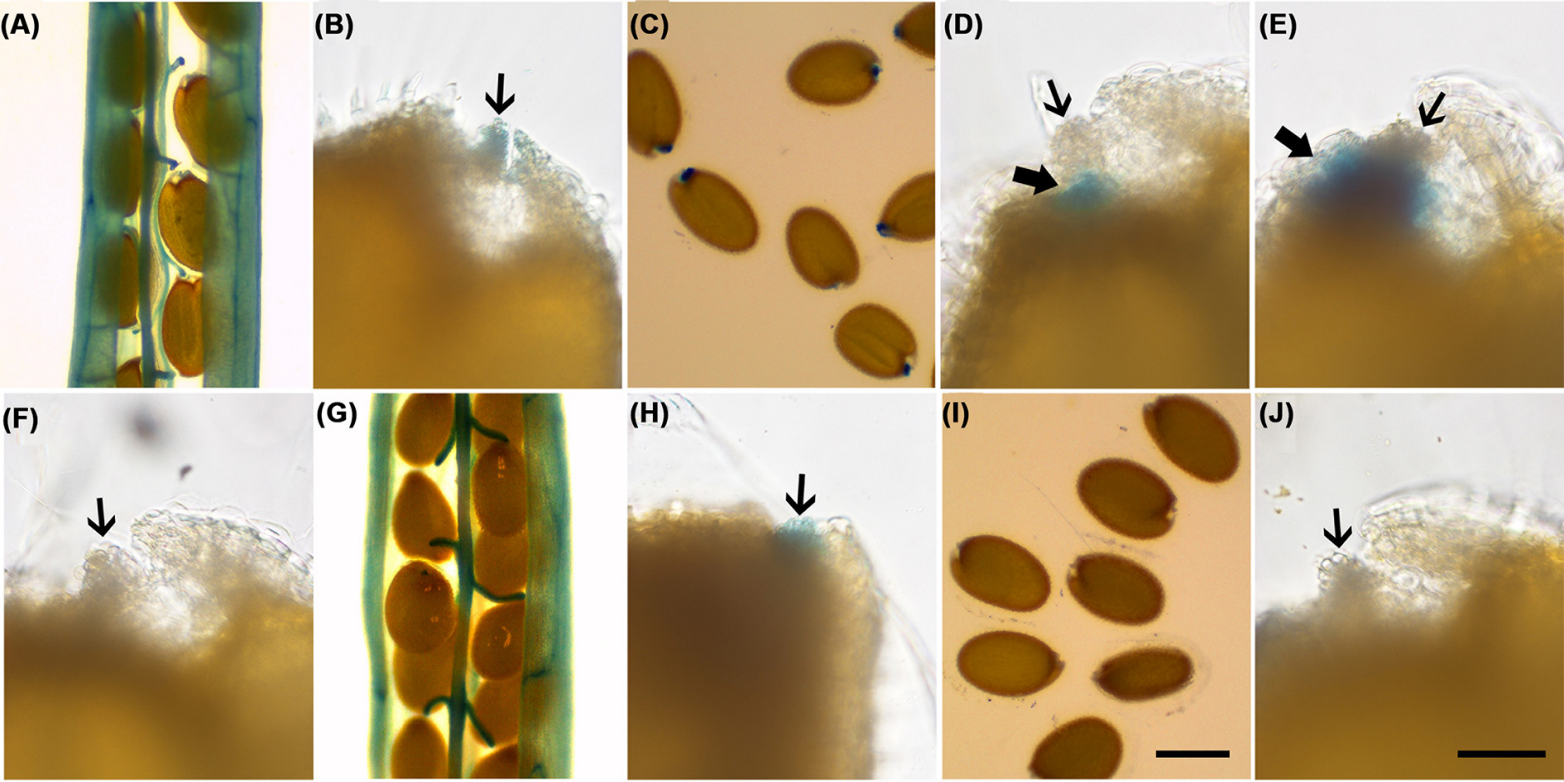
GUS staining patterns in dehisced siliques and postharvest seeds in *AFB1:GUS* and *AFB5:GUS* lines (**A–F**) *AFB1:GUS*. (**G–J**) *AFB5:GUS*. (A) A T_1_ silique. (B) An abscised T_2_ seed in (A), showing GUS signal in the hilum (arrow). (C) Postharvest dry T_2_ seeds directly placed in GUS staining solution. (D) A seed in (C), showing the GUS signal around the hilum (thick arrow). The hilum was GUS-signal-free (thin arrow). (E) A T_2_ seed that was imbibed on the MS agar medium for 6 h before GUS staining. (**F**) A T_2_ seed that was imbibed on an MS agar medium for 10 h before GUS staining solution. (G,H) GUS signals in a T_1_ silique and a T_2_ seed from the silique, respectively. (I,J) Postharvest dry seeds were subjected to the same treatment as in (C) and (D). Scale bar in (I) for (A), (C), (G) and (I) = 400 µm, and scale bar in (J) for the remaining images = 50 µm.

To seek clues on how AFB1 and AFB5 regulate seed germination, their expression patterns in nearly mature (slightly yellow) fruit and the postharvest dry seeds during imbibition were determined using the *AFB1:GUS* and *AFB5:GUS* reporter lines. Expression signals of *AFB1* and *AFB5* were observed to be in the vasculature and other cells in the fruit wall and the funiculus (Figure 7A,B,G,H). Placing dry *AFB1:GUS* seeds directly into the GUS staining solution induced its expression in a small seed coat region surrounding the hilum approximately 4 h later (Figure 7C,D). The expression was also detected if the seeds were first imbibed on the MS medium for six hours before the GUS staining process (Figure 7E) but it was not so if the seeds were imbibed on the MS medium for 10 hours before the GUS staining process (Figure 7F). However, no such expression was detected with the *AFB5:GUS* seeds (Figure 7I,J). These results indicated that *AFB1* and *AFB5* had overlapping expression domains in the fruit and *AFB1*, not *AFB5*, was also transiently expressed in the seed coat region surrounding the hilum in the early phase of imbibition. Based on the maternal inhibitive effects of AFB1 and AFB5 on seed germination and their expression patterns in the maternal tissues, it is likely that AFB1 and AFB5 act in the funiculus and the seed coat region surrounding the hilum to regulate seed dormancy.

## Discussion

### The absence of previous reports on the seed germination phenotypes

*AFB1* and *AFB5* transgenic lines and *afb1* and *afb5* mutants have been studied before, but no seed germination phenotypes have been reported to occur in these plants [28,32]. However, the lack of previous reports on the seed germination phenotypes should not come as a surprise. T_1_ seeds of *AFB1* and *AFB5* transgenic plants do not have a germination defect because their effects on seed germination are maternal. Laboratories would have had no difficulty isolating the transgenic plants. In our investigation, at the T_2_ generation, most of the transgenic lines had moderate differences or no difference in seed germination when compared with the wild-type. We used seeds that were dried and stored at room temperature, which likely helped detect the differences between the test and the control seeds since seed stratification or storage at a low temperature generally promotes Arabidopsis seed germination. The results of our experiments as shown in Figure 3C,B also demonstrate that the timing of seed counting is critical to determining the differences in germination frequency between the test and control seeds. These factors can potentially be the reasons for overlooking the seed germination phenotypes associated with either *AFB1* and *AFB5* transgenic plants or *afb1* and *afb5* mutants.

### Pre- and postharvest inhibition of seed germination by auxin signaling

Our findings in this report indicate that *AFB5* is expressed in maternal tissues at the preharvest stage and yet AFB5 plays a role in inhibiting postharvest seed germination. The most likely tissue in which AFB5 exerts this effect is the funiculus since it directly connects with the seed coat and is the only passage of regulatory molecules from the maternal tissues to the seed. *AFB1* is expressed in a similar pattern compared with that of *AFB5* at the preharvest stage, suggesting that AFB1 may overlap with AFB5 in inhibiting postharvest seed germination. However, because *afb1* and *afb5* single mutants exhibited faster germination than the wild-type, AFB1 or AFB5 is not functionally redundant with any other member of the TIR1/AFB family. Furthermore, because *afb1-5* and *afb5-5* seemed to have a synergistic effect in promoting seed germination based on the comparison between the phenotype of the *afb1-5afb5-5* double mutant and those of the corresponding single mutants, AFB1 and AFB5 may act in a complex netwok in regulation of seed germination. Other scenarios, such as AFB1 and AFB5 acting in the same pathway or two parallele pathways in regulation of seed germination, would have resutled in the double mutant phenotype either resembling one of the single mutant phenotypes (epistasis) or being additive of the single mutant phenotypes.

An intriguing finding in the present study is that *AFB1* was also transiently expressed in a small seed coat region surrounding the hilum during the early phase of imbibition, whereas *AFB5* was not. This observation suggests that AFB1 plays an additional role in inhibiting postharvest seed germination, which may be translated into a greater effect from AFB1 than from AFB5 in this regulation. Consistent with this possibility, some *AFB1* transgenic lines had 100% non-germination of T_2_ seeds while no *AFB5* transgenic T_2_ line had such a severe defect (Figure 1). The expression patterns of *AFB1* and *AFB5* in the maternal tissues also suggest that the entrance into the seed, may it be the funiculus, the hilum, or the seed coat region surrounding the hilum, is a key location for controlling seed germination.

### Divergence and integration of signaling pathways for seed germination

Our findings in this report demonstrate two features of regulation of seed germination by auxin. On the one hand, auxin signaling can strongly inhibit seed germination as demonstrated by the complete or nearly complete non-germination phenotypes of some of the *AFB1* and *AFB5* transgenic lines. On the other hand, this negative regulation of seed germination by auxin seems to be moderated by functional divergence between AFB1 and AFB5 and by transient expression of *AFB1* during early imbibition. Moderation in the effect of a signaling pathway may be especially relevant to seed germination as seed germination is expected to occur through integration of multiple positive and negative signals. Plants have presumably evolved complex, sensitive, and yet flexible regulatory mechanisms in controlling seed germination. Moderation in the effect of any one particular signaling pathway on seed germination may be a necessary feature in a network structure whose output is not dominated by one signaling input. Multiple signaling pathways involving auxin, ABA, and GA have been found to regulate seed germination in a negative or positive manner [1,3–5,7]. Understanding how these signaling pathways integrate in seed germination regulation requires the identification of germination executors onto which the positive and negative signaling pathways converge.

### Abundance of AFB1 an AFB5 in reproductive tissues

The 15 F-box proteins identified in this investigation represent the largest number of F-box proteins identified so far in Arabidopsis using the ASK1 protein as the bait (Supplementary Table S1). For comparison, Risseeuw et al. [33] identified 21 F-box proteins that interact with ASK1 by the yeast two-hybrid assay using a cDNA library prepared from Arabidopsis 1- to 4-week old seedlings [34]. The yeast two-hybrid study did not find that SKIP16 interacts with ASK1 but with ASK2. In fact, only ATPP2-B11 and SKIP22 were found in both Risseeuw et al’s and our investigations. These differences could result from the technical and/or plant material differences between the two investigations. However, both investigations identified only a small fraction of the SCF components [35,36]. It is likely that both the mRNAs and the proteins are expressed at low levels for most of the F-box protein genes so that they were not detected in these investigations. If so, our results would suggest that AFB1 and AFB5 are more abundant than the other AFBs in the reproductive tissues. Consistent with this possibility, Prigge et al. [32] found that the transcripts of AFB1 and AFB5 were higher than those of the other AFBs in the reproductive tissues.

## Materials and methods

### Plant materials and growth conditions

The *Arabidopsis thaliana* accession Columbia-0 (Col-0) was used as the wild type. Three T-DNA insertion mutant lines, *afb1-3* (SALK_070172C) [27,37], *afb1-5* (SALK_144884C) [38], and *afb5-5* (SALK_110643) [32] were obtained from the Arabidopsis Biological Resource Center (Ohio State University, Columbus, OH, U.S.A.). The *afb1-5afb5-5* double mutant plants were isolated from the F_2_ population of a cross between *afb1-5* and *afb5-5*. The genotypes of the single and double mutants were verified by PCR (Supplementary Table S2 and Figure S1). All the transgenic lines were generated in either the *afb1-3* or *afb5-5* background, except the GUS lines that were in the Col-0 background.

All seeds were air dried at room temperature for at least four weeks and stored at room temperature. Seeds used in an experiment were harvested from plants grown at approximately the same time and in the same growth chamber and were younger than 3 years old. The growth chamber was set at 22°C or 20°C (GA experiment only) with a daily light regime of 16 h fluorescent light supplemented with tungsten light (light intensity = ∼50 µmol·m^−2^·s^−1^ near the soil or Petri dish surface) and 8 h darkness. Sunshine MVP growing mix (Sungro Horticulture, Agawam, Massachusetts, U.S.A.) was used for soil-grown plants. The medium for seed germination and plant growth contained 4.3 g/L Murashige and Skoog (MS) salt base (Gibco, Waltham, MA, U.S.A.), 1% (w/v) sucrose, and 1% (w/v) agar. For testing plants’ sensitivity to IAA, the same agar medium plate was supplemented with 0.1 µM IAA. Seeds were sterilized with bleach before they were planted on the agar medium. To grow plants on the surface of the agar medium, the Petri dishes were let stand on their edges. For quantifying seed germination frequency, a piece of filter paper (90 mm diameter) was first moistened with 2 ml double-deionized water in the lid of a Petri dish (100 mm × 15 mm) before the seeds of a genotype were sprinkled onto the filter paper. The seeds were then covered with the bottom of the Petri dish. The Petri dish was incubated upside down in the growth chamber.

### Gene constructs and generation of transgenic plants

The *ASK1:ASK1-FLAG* gene construct contained the same promoter and coding region as described before [39] with the FLAG tag fused at the C-terminus. The same *ASK1* promoter was used in the *ASK1:AFB1* and *ASK1:AFB5* gene constructs that also contained either an N- or C-terminal FLAG. The *AFB1:AFB1* constructs contained a 1084-bp promoter fragment, the coding region of *AFB1*, a FLAG tag at either the N- or C-terminus, and a 418-bp fragment after the stop codon. The same *AFB1* promoter region was used for *AFB1:GUS*. The *AFB5:AFB5* constructs contained a 934-bp promoter fragment, the coding region of *AFB5*, a FLAG tag at either the N- or C-terminus, and a 478-bp fragment after the stop codon. The same *AFB5* promoter region was used for *AFB5:GUS*. The PCR primers corresponding to the specified genomic or plasmid regions with appropriate flanking restriction enzyme sites were used to amplify and clone the fragments. pGEM®-7Zf(+) (Promega, Madison, WI, U.S.A.) was used as an intermediate vector and pPZP121 the final vector [40]. Plant transformation was carried out with the Agrobacterial strain GV3101 as previously described [40].

### Co-immunoprecipitation and liquid chromatography-tandem mass spectrometry

In these experiments, we used a transgenic line containing *ASK1:ASK1-FLAG* as the source of plant tissues. This transgenic line is the original *ask1-1* mutant complemented with the *ASK1:ASK1-FLAG* transgene. The *ask1-1* mutant is a null allele that does not produce the *ASK1* transcript [41]. We reasoned that Co-IP using this line will be more efficient than the transgene in the wild-type *ASK1* background because of the absence of the competition from the endogenous ASK1. Co-IP was performed with an anti-FLAG antibody and cell lysates from inflorescences with closed buds and young flowers. The procedure for the Co-IP experiments was as previously described [42]. The CoIPed proteins were first separated in an SDS-PAGE gel and slices of the gel covering the full range of protein sizes were subjected to trypsin digestion followed by LC/MS/MS for protein identification using an LTQ-Orbitrap XL hybrid mass spectrometer [43,44]. The LC-MS/MS RAW files were used for database searching via the software applications Mascot (Matrix Science) and X! Tandem (www.thegpm.org). Search results were visualized and analyzed using the software Scaffold. The experiments were independently conducted four times.

### Quantification of gene transcripts

The mRNA samples were extracted using the RNeasy Plant Mini kit (Qiagen, Valencia, CA, U.S.A.) from 2-week-old seedlings grown on the MS agar medium. The reverse transcription and qPCR were carried out as previously described [45]. For each genotype, three biological replicates and three technical replicates were performed.

### Characterization of mutant phenotypes

Surface-grown seeds or seedlings in the Petri dishes were photographed using a Canon camera (PowerShot SX170 IS) on a copy stand five or six days after planting. Other micrographs were taken on a Nikon dissecting microscope (SMZ1000) or a Nikon compound microscope (Eclipse 80i) equipped with a digital camera (DS-Ri1) using NIS Elements (BR 4.40.00).

To determine the seed germination frequency of a genotype, seeds were put on the water or 1-µM-GA (Quick-Dissolve™, GoldBio, St Louis, MO, U.S.A.)-moistened filter paper in an upside down Petri dish incubated in a growth chamber. Germinated and ungerminated seeds were scored under a dissecting scope at the times of 58- and 72-h imbibition with water or at the times of 46- and 64-h imbibition with the GA solution. Seed germination frequency was calculated as the percentage of germinated seeds out of the total seeds scored. A seed is considered germinated when the seed coat was broken and part of the white radicle, no matter how small it was, could be seen. When necessary, a seed was gently flipped over with a fine syringe needle to confirm whether the emergence of the radicle was on the underside of the seed. More than 100 seeds were counted to arrive at each germination frequency value and three or more lines for each genotype were tested two or more times.

### β-Glucuronidase (GUS) staining

Incubation of samples for GUS staining was at 37°C for 24 h in the solution as previously described [45]. Three or more transgenic lines were tested and results from representative ones were used for Figure 7.

### Image manipulation

Composite images were created in Adobe Photoshop CC 2014. The entire photos were subjected to modest adjustments in brightness and contrast in Adobe Photoshop. Plots in Figures 3 and  4 were first created in Excel and then copied and pasted into Adobe Photoshop.

## Supplementary Material

Supplementary Figure S1 and Tables S1-S2

## Data Availability

All relevant data can be found within the manuscript and its supporting materials.

## Abbreviations

ABS: abscisic acid
AFB: AUXIN SIGNALING F-BOX
ARF: AUXIN RESPONSE FACTOR
Co-IP: co-immunoprecipitation
GA: gibberellic acid
GUS: β-glucuronidase
IAA: indole acetic acid
