# Supplementary material for: Negative regulation of seed germination by maternal AFB1 and AFB5 in Arabidopsis
Source: Biosci Rep. 2022 Sep 12;42(9):BSR20221504. doi: 10.1042/BSR20221504 (PMC9469108; doi:10.1042/BSR20221504)
Supplement: Supplementary Figure S1 and Tables S1-S2 [file BSR-2022-1504_supp.pdf]

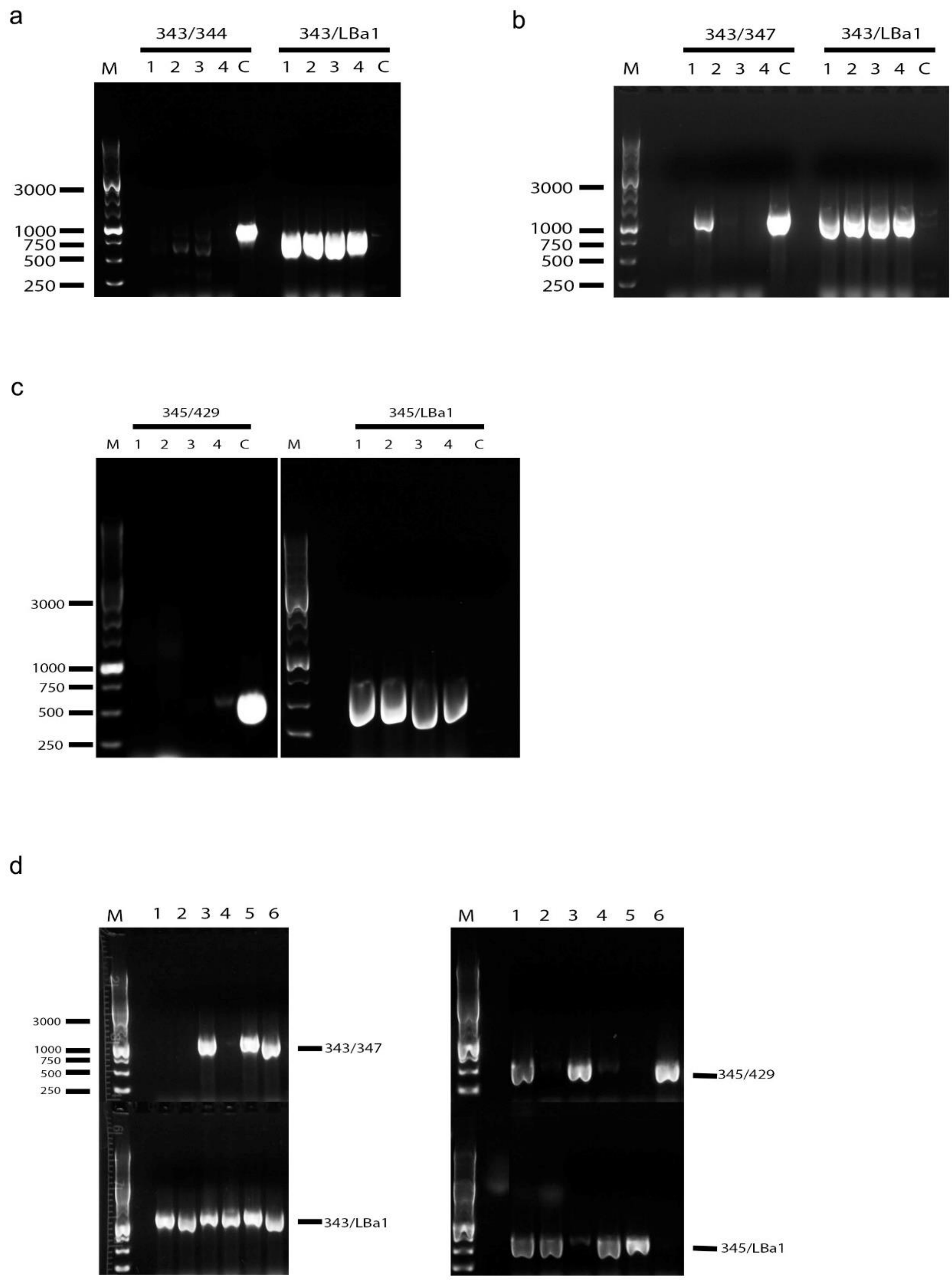

Figure S1. Identification of the *afb1* and *afb5* single mutants and *afb1-5afb5-5* double mutant by PCR. (a) Plant 1-4 were the *afb1-3* mutant. (b) Plant 1, 3, and 4 were the *afb1-5* mutant. (c) Plant 1-3 were the *afb5-5* mutant. (d) Plant 2 and 4 were the *afb1-5afb5-5* double mutant. M, DNA ladder; and C, non-transgenic control.

Table S1. SCF components identified by Co-IP and LC-MS/MS in four independent experiments

Protein	Times identified	Biological function
ASK1	4	Many functions
CUL1	3	Many functions
F-box proteins		
AFB1	4	Auxin signaling
AFB5	4	Auxin signaling
SKIP16	4	Possibly in carbohydrate transport and metabolism
COI1	3	Wound/jasmonate-induced transcriptional regulation
At5g06550	2	Unknown function; with a JmjC domain
At4g00755	2	Unknown function; with a conserved domain
ATPP2-B11	1	Carbohydrate binding, phloem protein
SKIP22	1	Unknown function, homologous to yeast Met30p
AFB2	1	Auxin signaling
At1g27540	1	Unknown function
A11g25141	1	Unknown function
At1g67190	1	Unknown function
At1g13570	1	Unknown function
At1g25150[a]	1	Unknown function
At1g64840	1	Unknown function; with a conserved domain

[a]At1g25150 is identical in amino acid sequence to other four proteins encoded by closely situated genes At1g25055, At1g24881, At1g25211, and At1g24800 on chromosome 1.

Table S2. Primers used in genotyping the *afb1* and *afb5* single mutants and *afb1-5afb5-5* double mutant in qPCR

	Primer	Sequence
For genotyping	343	5’-GTGAACTGGACACAGAAGTAGAG-3’
	344	5’-GGTGACTGATGAGTGCTTAGAG-3’
	345	5’-CTCCGGCATCAACTCTCTAAAC-3’
	347	5’-TTATGGGTCTCCGATTCCCACCTAA-3’
	429	5’-GCTCCTTGGGTTTCTACTATGG-3’
	LBa1[a]	5’-TGGTTCACGTAGTGGGCCATCG-3’
For qPCR	443	5’-AAGCTATGGGTGATGGACTTGAT-3’
	444	5’-AAGATCTGGCTCAGATGGAAACA-3’
	431	5’-TGTTCTGGGCCCTTGATTCAAT-3’
	432	5’-CACTGTCTTCCCGAGGATCAAA-3’

[a] LBa1, T-DNA left-border primer.
